# Tendency towards clonality: deviations of meiosis in parthenogenetic Caucasian rock lizards^†^

**DOI:** 10.1093/biolre/ioaf091

**Published:** 2025-05-22

**Authors:** Victor Spangenberg, Marine Arakelyan, Sergey A Simanovsky, Yana Dombrovskaya, Emma R Khachatrian, Oxana Kolomiets

**Affiliations:** Scientific Center of Zoology and Hydroecology NAS RA, P. Sevak 7, Yerevan 0014, Armenia; Vavilov Institute of General Genetics RAS, Gubkin 3, Moscow 119991, Russia; Faculty of Biology, Yerevan State University, Alex Manoogian 1, Yerevan 0025, Armenia; Severtsov Institute of Ecology and Evolution RAS, Leninsky 33, Moscow 119071, Russia; Scientific Center of Zoology and Hydroecology NAS RA, P. Sevak 7, Yerevan 0014, Armenia; Severtsov Institute of Ecology and Evolution RAS, Leninsky 33, Moscow 119071, Russia; Scientific Center of Zoology and Hydroecology NAS RA, P. Sevak 7, Yerevan 0014, Armenia; Vavilov Institute of General Genetics RAS, Gubkin 3, Moscow 119991, Russia

**Keywords:** reticulate evolution, meiosis, synaptonemal complex, oogenesis parthenogenesis, genome duplication, bivalent, multivalent, Muller’s ratchet, recombination

## Abstract

Cytogenetic mechanisms of unisexuality in diploid parthenogenetic species of the genus *Darevskia* have remained debatable until recently. The mechanism that allows the unisexual form to maintain heterozygosity in a number of generations is important for its long-term existence in nature. In this work, for the first time, for parthenogenetic species of the genus *Darevskia*, in addition to primary oocytes with the usual ploidy (18 + ZW bivalents in meiotic prophase I), oocytes that underwent premeiotic genome endoduplication and carried a doubled number of bivalents (36 + ZZ + WW) were found. Here, we present a detailed comparative analysis of the preparations of synaptonemal complexes in oocyte nuclei without and with genome endoduplication and the behavior of sex Z and W chromosomes. We show the details of the assembly of bivalents in pachytene nuclei, where either homeologs or doubled identical copies of chromosomes compete for synapsis and form multivalents. For the first time, the WW sex bivalent has been visualized in parthenogenetic reptiles. We show the reverse side of meiotic deviations in obligate parthenogenesis—cases of nonviable embryos with specific abnormalities.

## Teaser

The cytogenetic mechanism of unisexuality in a parthenogenetic lizard of the genus Darevskia was elucidated. Prophase I pachytene oocytes after premeiotic genome endoduplication were identified. This work demonstrates the remarkable plasticity of meiotic prophase I in this parthenogenetic lizard.

## Graphical Abstract

**Graphical Abstract:**
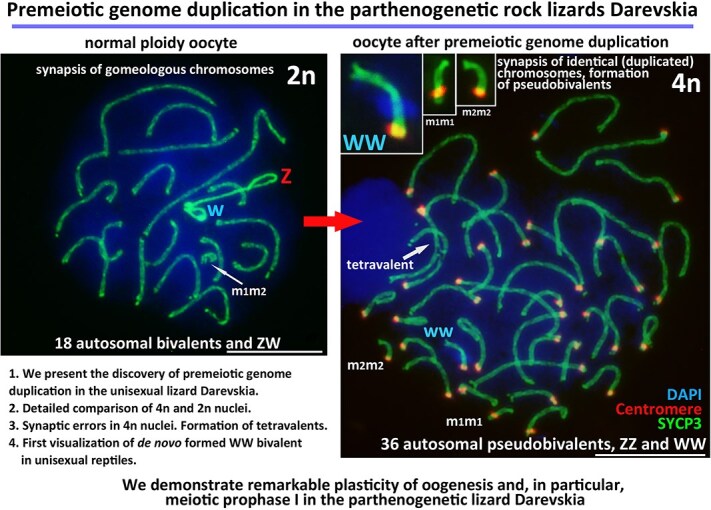

## Introduction

Studies of reticulate evolution and parthenogenesis in reptiles over the past decades have revealed several important characteristics of the biology of this large and highly diverse taxonomic group.

First, intensive interspecific hybridization occurs in reptiles, which can lead to the formation of parthenogenetic forms [1–4]. Phylogenetic relationships between gonochoristic parental species and hybrid parthenogenetic forms have been established for most of these reptile taxa [5–11]. It is assumed that a certain level of divergence and, as a consequence, the incompatibility of parental karyotypes in interspecific hybrids can lead to the start of a unisexual mode of reproduction, i.e. a new parthenogenetic lineage [12, 13]. The combination of two divergent genomes in one hybrid karyotype of a parthenogenetic species is associated with a number of deviations at the key stage of the life cycle—meiosis. Parthenogenesis is supposed to be a way to avoid hybrid sterility [14].

Second, there are obligate parthenogenetic forms of reptiles of different ploidy levels, namely, diploids [2], the most common triploids [15], and tetraploids [16, 17]. Within the group of Caucasian rock lizards, only diploid parthenogenetic forms, which initially appeared as hybrids, have been preserved as independent species [18].

Third, cytogenetic studies of interspecific hybrids revealed an important feature of gametogenesis in reptiles—weak checkpoints of meiosis. In particular, the germ cells of hybrid triploid males of the genus *Darevskia* successfully pass through both divisions of meiosis [19]. These triploid hybrids result from the mating of parthenogenetic species with their sexual relatives in hybrid zones. Numerous defects in chromosome synapsis and incomplete DNA double strand breaks (DSB) repair did not lead to arrest of meiosis or spermatogenesis. We assumed that there was weak selection of meiocytes with abnormalities in prophase I [19].

Fourth, molecular biological studies of distinct populations of parthenogenetic species of hybrid origin indicate the possibility of a *de novo* appearance of minor differences between them [20–22]. In addition, we identified evolutionary changes in the karyotypes of parthenogenetic forms [23, 24]. Overall, it appears that due to the plasticity of meiosis and its weak checkpoints, some diversity of obligate unisexual forms of hybrid origin may arise in the genus *Darevskia*.

From the point of view of the cytological mechanisms of unisexual reproduction, several variants of automixis have been identified [25]. Each variant has different consequences for offspring [26]. Two key preconditions for the formation and long-term existence of the unisexual form are (a) a successful combination of parental genomes and (b) the presence of a reliable mechanism for maintaining the primary heterozygous state of the F1 hybrid in a number of generations [5].

The most stringent conditions for maintaining parental karyotypes in the parthenogenetic form in an unchanged (original) state can be provided by the mechanism of premeiotic genome endoduplication. In this case, in a hybrid karyotype consisting of pairs of homeologous chromosomes, each chromosome is duplicated before meiosis. During the early stages of meiotic prophase I, in the leptotene and zygotene stages, synapsis occurs not between hom*e*ologs but between just appeared two identical chromosomes [27]. The assembled synaptonemal complexes (SCs) in this case are called “pseudobivalents”. Thus, the number of pachytene “pseudobivalents” in the nucleus after genomic endoduplication will be two times greater than usual. This mechanism allows both divisions of meiosis to occur, avoiding the problems of chromosome incompatibility between two divergent parental species [14, 15, 27]. A study was recently published that describes the later stages of meiotic prophase I, lampbrush chromosomes, in parthenogenetic Darevskia lizards. However, the preceding stages of meiosis were not studied. The authors describe exclusively doubled sets of chromosomes in the nuclei of primary oocytes [28].

Despite the well-known general scheme of this cytological mechanism in reptiles, some details of prophase I of meiosis in nuclei with genomic endoduplication remain elusive. This is due to the difficulties in obtaining rare endoduplicated oocytes I in the pachytene and early diplotene stages. These stages allow visualization of competitive interactions between chromosomes and deviations from the canonical scheme of meiotic prophase I.

Previous studies of hybrid diploid karyotypes of *Darevskia* parthenogenetic species have shown the preservation of the species-specific composition of pericentromeric chromosome regions [24, 29]. That is, half of the chromosomes bear the pericentromeric satellite DNA of the paternal species, whereas the second half, including the W chromosome, has the maternal composition of pericentromeric satellite DNA. However, a more detailed study using the CGH approach to determine the somatic karyotype of the parthenogenetic species *D. rostombekowi* revealed species-specific differences not only in pericentromeric regions but also in the interstitial regions of homeologous chromosomes [23, 24]. These data indicated the possibility of maintaining the original state of the chromosomal sets of parental species in *Darevskia* unisexual species through many generations. Therefore, the clarification of the mechanism of ploidy restoration during meiosis and maintenance of heterozygosity in *Darevskia* species has been debated and requires further detailed study [18].

Previously, we suggested that the restoration of ploidy in unisexual species of *Darevskia* occurs by automixis according to the central fusion variant. That is, a result of a specific postmeiotic fusion of products of meiosis [29]. Oocytes I with normal ploidy was assumed to form a successful synapsis of hom*e*ologous (but not homologous) chromosomes. Thus, samples obtained from two populations of *D. unisexualis* demonstrated numerous pachytene and early diplotene nuclei in oocytes I with normal ploidy and assembled autosomal bivalents [24, 29]. No significant deviations in the assembly of homeologous bivalents were detected. These results contrast strongly with those of other studied parthenogenetic reptiles, where hom*e*ologous synapsis is fragmentary only: peritelomeric synapsis, partial synaptic zones, and the formation of multivalents. The assembly of bivalents in such oocytes terminates at the zygotene stage [15]. This difference is apparently due to significant divergence in parental chromosomal sets or because of the more stringent synaptic checkpoints [14, 15].

In this work, we conducted a detailed study of meiosis in oocytes I of the unisexual species *Darevskia armeniaca* to elucidate the characteristics of meiotic prophase I, which allows long-term occurrence of the unisexual form.

## Materials and methods

### Animals studied

*D. armeniaca* is a diploid parthenogenetic species of hybrid origin that results from an ancestral cross between the paternal species *Darevskia valentini* and the maternal species *Darevskia mixta* [30]. Three juvenile individuals and one adult were used in the study. All the experiments were approved by the Ministry of Environment Republic of Armenia, permission No. 3/29.7/1043, in accordance with the Regulations for Laboratory Practice.

### Isolation of primary oocytes

Primary oocytes were obtained from two juvenile *D. armeniaca* Tsahkadzor population (specimen VSj0401; specimen VSj0405) 10 days after hatching. Preparations of SCs from oocytes I were obtained according to Spangenberg (2022) [31]. No colchicine treatment was applied before obtaining the preparations of primary oocytes.

### Immunostaining

The slides were washed with phosphate-buffered saline (PBS) and incubated with primary antibodies diluted in antibody dilution buffer (3% bovine serum albumin (BSA) and 0.05% Triton X-100 in PBS) at 4°C overnight. Axial elements of meiotic chromosomes were detected using rabbit polyclonal antibodies against the protein SYCP3 (ab15093, 1:250; Abcam, Cambridge, UK), centromeres were detected using the human anti-centromere antibodies ACA (1:500; Antibodies Incorporated 15–234; Davis, CA, USA) and antibodies against DNA mismatch repair protein MLH1 (1:250; Abcam) was used for the detection of the late recombination sites. After washing, the following corresponding secondary antibodies diluted in ADB: goat anti-rabbit immunoglobulins (Ig)G, Alexa Fluor 488 (ab150077, 1:500; Abcam, Cambridge, UK), goat anti-human Alexa Fluor 555 (A21433; Thermo Fisher Scientific, Waltham, MA, USA) and goat anti-mouse Alexa Fluor 555 (1:500; Invitrogen, Carlsbad, CA, USA). Secondary antibody incubations were performed in a humid chamber at 37°C for 3 h.

All preparations were mounted with Vectashield Antifade mounting medium supplemented with 4′,6-diamidino-2-phenylindole (DAPI; Vector Laboratories, Burlingame, CA, USA).

### Mitotic chromosome preparation, C-banding and telomere repeats FISH mapping

Mitotic chromosome preparations were obtained from one juvenile and one adult *D. armeniaca* from Tsahkadzor population (specimen VSj0404; specimen VS404) from bone marrow and all somatic abdominal organs using a direct suspension technique [32]. C-banding was performed according to Salvadori et al. [33]. *In situ* hybridization with telomeric sequences was performed using 5′-TAMRA-(CCCTAA)4 (Syntol, Moscow, Russia) following a standard protocol [23].

### Microscopy

Meiotic preparations were examined and analyzed in October 2022 using an Axio Imager D1 microscope (Carl Zeiss, Germany) equipped with an AxioCam HRm CCD camera (Carl Zeiss, Germany), Carl Zeiss filter sets (FS01, FS38HE, and FS43HE), and image-processing AxioVision Release 4.8 software (Carl Zeiss, Germany). Meiotic bivalent lengths were measured using ImageJ software (https://imagej.net/ij/, accessed on 25 Feb 2025). The mitotic chromosomes were stained with DAPI, examined using a Leica DM microscope equipped with an Axiocam HRm CCD camera and filter set A, and processed with AxioVision Release 4.8. software (Carl Zeiss, Germany). The mitotic chromosome slides, conventionally stained with Giemsa, were examined using an Axioplan 2 Imaging microscope (Carl Zeiss, Germany) equipped with a CV-M4 + CL camera (JAI, Kanagawa, Japan) and Ikaros software (MetaSystems, Altlussheim, Germany). Meiotic prophase I stages were identified by analyzing the combination of basic morphological criteria used in the meiotic cell studies [34]. The rock lizards-specific features of the prophase I stages have been described by us before [35].

## Results

### Primary oocytes with normal ploidy *in D. armeniaca*

**Figure 1:**
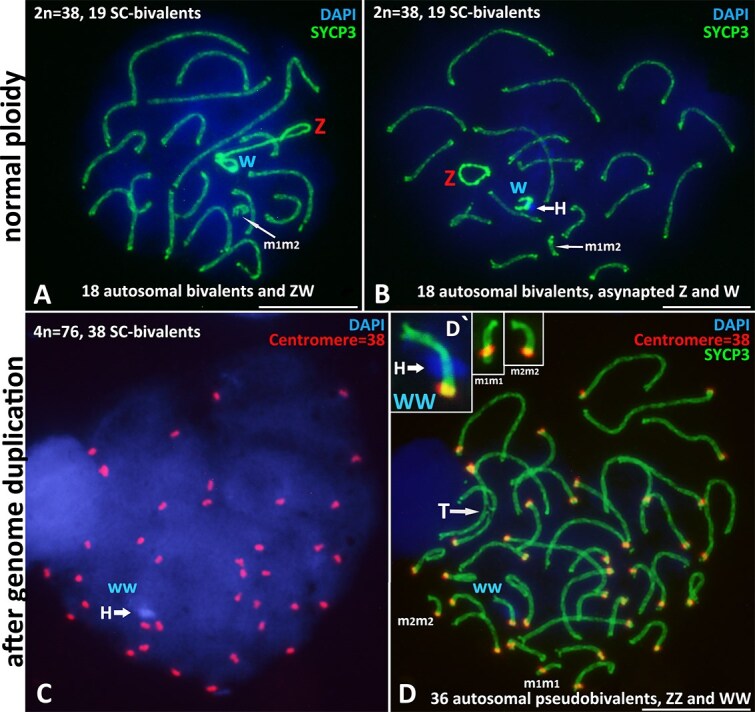
*D. armeniaca* oocyte I nuclei of normal ploidy (A–B) and after premeiotic genome endoduplication (C–D). Preparations of SCs of *D. armeniaca* 2n = 38 in early diplotene. Axial elements were immunostained with anti-SYCP3 antibodies, centromeres with ACA antibodies, and chromatin stained with DAPI 4′,6-diamidino-2-phenylindole. (A) Early diplotene nucleus of normal ploidy with 18 autosomal homeologous bivalents and the sex ZW bivalent. (B) Early diplotene nucleus of normal ploidy with 18 autosomal homeologous bivalents and sex Z and W univalents located separately; a heterochromatin block on the W-univalent is indicated with a white arrow. (С and D) Endoduplicated oocyte I nucleus with 38 pseudobivalents, sex WW-bivalent, containing a heterochromatin block (white arrow). Autosomal tetravalent are indicated with “T”. (D′) Enlarged fragments with WW-bivalent, and autosomal micro bivalents m1m1, m2m2. Scale bars: 10 μm.

From the primary oocytes of *D. armeniaca*, we obtained 118 pachytene and early diplotene spreads of SCs. Most of the oocytes I nuclei analyzed in this study had a normal number of bivalents 18A + Z + W, where A - autosomal bivalents. This is the usual number of bivalents for 2n = 38 diploid karyotypes in unisexual and gonochoristic *Darevskia* species (Figure 1A). We specifically separated the sex chromosomes in the record (Z + W) since the Z and W chromosomes in 53% of the studied nuclei (among 81 nuclei with detectable sex univalents) did not form a normal sex bivalent (without a region of local synapsis—pseudautosomal region, i.e. without PAR) and were not even located nearby (Figure 1B). In contrast, contact between Z and W sex chromosomes was found in only 47% of the oocyte I nuclei. The region of close proximity between Z and W was located within their centromeric regions (Figure 1A; Supplementary Figure SM1).

An important characteristic of the W sex chromosome in *D. armeniaca* is the interstitial block of heterochromatin, which is clearly visible via DAPI (Figure 1B and C) and Giemsa staining (Supplementary Figure SM2A). We detected this interstitial chromatin block using a C-banding approach (Supplementary Figure SM2 2B). The interstitial chromatin block on the W sex chromosome is an important characteristic that distinguishes it from autosomes (in particular, from the nearest pair of microchromosomes, m1 and m2). Telomere FISH mapping revealed no notable enrichment of telomeric repeats on the W chromosome (Supplementary Figure SM2С).

Finally, in non-duplicated oocyte nuclei we detected loading of the MLH1 protein (mismatch-repair protein) in meiotic bivalents in all the nuclei after immunostaining with anti-MLH1 antibodies. Z-stacking with three optical layers demonstrates at minimum 23 MLH1 foci per primary oocyte nucleus (Figure 3A(I)-A(II)-A(III)).

### Primary oocytes after genome endoduplication in *D. armeniaca*

**Figure 2:**
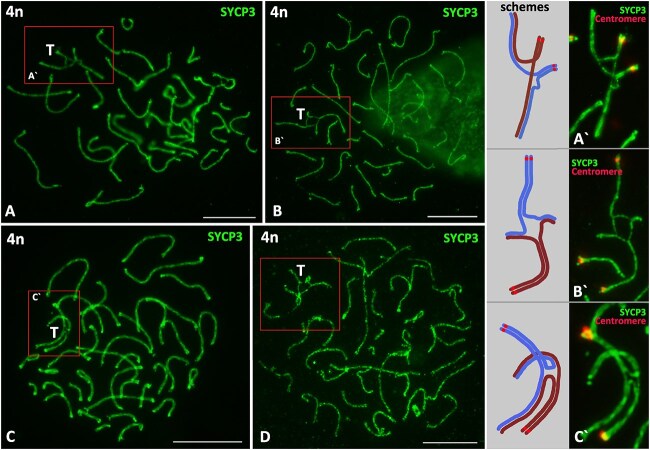
Formation of SC-tetravalents during competitive assembly of pseudobivalents and homeologous bivalents in *D. armeniaca* oocytes I after premeiotic genome endoduplication. (A), (B), (C), (D) SC preparation with ectopic synapsis of duplicated/homeologous chromosomes leading to formation of tetravalents indicated “T”. (A′), (B′), (С′) Fragments of SC preparation and schemes of tetravalents. SC axial elements immunostained with anti-SYCP3 antibodies; and centromeres immunostained with ACA antibodies. Scale bars: 10 μm.

In addition to numerous oocyte nuclei with normal ploidy, in both animals studied we found 19 endodouplicated nuclei with a doubled number of bivalents (36A + ZZ + WW, i.e. 38 pseudo bivalents) between pairs of newly duplicated chromosomes (Figure 1C and D, Figure 2). Such nuclei were detected at the pachytene (complete synapsis) and early diplotene (with desynaptic forks at the ends of bivalents) stages. We detailed important characteristics of the endoduplicated primary oocyte nuclei, which are listed below.

### Identification of the WW bivalent in the endoduplicated nuclei

For the first time in the studies of parthenogenetic reptiles, the WW bivalent was visualized in endoduplicated pachytene nuclei as the only bivalent carrying a well-defined interstitial heterochromatin block, which was clearly observed via DAPI staining (Figure 1C, D, and D′). In all the analyzed nuclei with endoduplication, we found this single bivalent with a heterochromatin block. This fact proves the assembly of the WW bivalent, namely, from the newly duplicated identical W chromosomes.

- *Ectopic synapsis and formation of synaptonemal complex tetravalents.*

**Figure 3:**
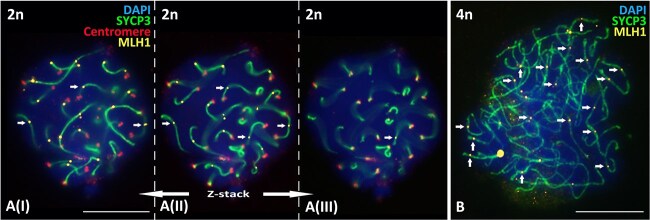
Immunodetection of meiotic recombination marker MLH1 in two types of oocytes of parthenogenetic species *D. armeniaca*. A(I)-A(II)-A(III) z-stacking of non-duplicated *D. armeniaca* oocyte nucleus. (B) Oocyte nucleus after genome endoduplication. Axial elements were immunostained with anti-SYCP3 antibodies, centromeres with ACA antibodies, mismatch repair sites in the structure of assembled bivalents are immunostained with anti-MLH1 protein antibodies (some of them are indicated by white arrows), and chromatin stained with DAPI 4′,6-diamidino-2-phenylindole. Scale bars: 10 μm.

In five oocyte nuclei with endoduplication, we found the formation of SC-tetravalents (Figure 1D, indicated “T”; Figure 2). The preparation of SCs with different configurations and details of these structures are presented in Figure 2. Pronounced synapsis was observed at the distal ends of the acrocentric chromosomes (Figure 3A–A′) and in the centromeric regions (Figure 3B–B′), and equal ectopic assembly was observed in the distal and centromeric regions (Figure 3C–C′).

- *Detection of crossing over marker, the MLH1 protein, in bivalents of enoduplicated D. armeniaca oocytes.*

Immunostaining with anti-MLH1 antibodies revealed loading of the mismatch repair protein MLH1 into the structure of meiotic bivalents in the endoduplicated oocyte nuclei and is shown in the Figure 3B.

**Figure 4:**
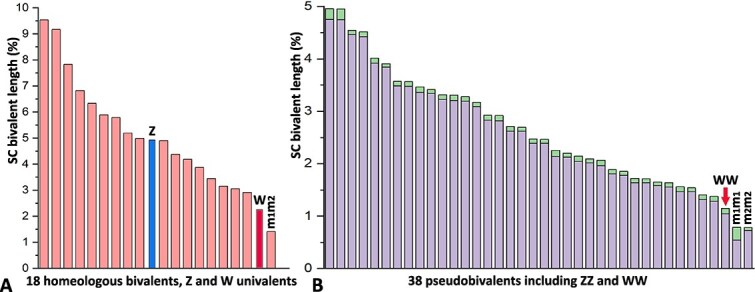
Ideograms of two types of oocytes of diploid parthenogenetic species *D. armeniaca*. (A) Normal ploidy oocyte with 18 bivalents of homeologous chromosomes and Z and W univalents. The positions of the centromeres are not indicated because of minor differences in the chromosomal arms of the homeologs. (B) Oocyte after genome endoduplication with 38 pseudobivalents (synapted copies of chromosomes 36A + ZZ + WW), q-arms, and p-arms. The WW-sex bivalent is indicated with a red arrow. ZZ-sex bivalent is indistinguishable from autosomal pseudobivalents and is not identified.

We performed detailed measurements of the preparation of SCs obtained from the normal ploidy and endoduplicated nuclei. Ideograms of the SC-karyotypes demonstrate the lengths of bivalents of the homeologs (Figure 4A) and pseudobivalents (Figure 4B).

- *Deviations in the development of embryos of the parthenogenetic species D. armeniaca.*

During the incubation of *D. armeniaca* eggs in our works, several of them stopped developing and two cases were recorded by us with large live embryos that failed to hatch from the eggs due to multiple developmental abnormalities, including unclosed body cavities, complete absence of the lower jaw, disproportion and curvature of the jaws. We presented photographs of these two embryos in the Supplementary Materials (Supplementary Figure SM3).

## Discussion

### Meiotic prophase I in oocytes without chromosome duplication

Most of the pachytene and early diplotene oocytes I we previously studied [24, 29] had surprising features for hybrid karyotypes; successful synapsis of all autosomal bivalents composed of pairs of hom*e*ologs but not homologous chromosomes and even loading of MLH1 protein in the meiotic bivalents (Figure 3A). The fact that we detected a homeologous but not homologous synapsis in oocytes without chromosome duplication was previously proven by us [24]. Visualization of the autosomal SC-trivalent in the unisexual form with an odd number of chromosomes (*D. unisexualis* 2n = 37) confirmed the synapsis of the hom*e*ologs [24]. Additional evidence for this was the significant divergence of centromeric DNA of the chromosomes involved in meiotic trivalent formation revealed by comparative genomic hybridization (CGH) [24].

Interestingly, in studies of diploid parthenogenetic species of the genus *Aspidoscelis* [15, 27], *Lepidodactylus*, *Hemiphyllodactylus* and *Heteronotia* [8], the authors noted that oocytes without genome duplication necessarily contain unpaired chromosomes—univalents. This pattern is typical for all studied diploid parthenogenetic species, except for unisexual species of the genus *Darevskia*. The authors justifiably assumed that such oocytes with long regions of asynapsis are doomed to undergo apoptosis due to the zygotene or pachytene checkpoints [8, 27]. Thus, cells with genome duplication are the only cells that are able to reach the diplotene stage [15].

In contrast, in two *Darevskia* parthenogenetic species we have studied to date (*D. unisexualis* and *D. armeniaca*), in addition to nuclei at the pachytene stage without visible synaptic disturbances, we also found a large number of normal diplotene nuclei [24, 29] and this study (Figure 1A and B, Supplementary Figure SM1A). This is an important feature of the unisexual *Darevskia* species. Theoretically, such cells with only hom*e*ologous synapsis could overcome the synaptic checkpoints of meiosis I, similar to what has been observed in the triploid hybrid individuals *D. unisexualis x D.valentini* we studied before [19].

It remains unclear whether such primary oocytes without endoduplication can undergo postmeiotic fusion, as suggested by our previous studies [24, 29]. If so, what contributions do they make to the offspring of unisexual species? Perhaps such oocytes are eliminated from the germ cell line and undergo apoptosis. It is important to recall the findings of previous studies and our own observations of the incubation of egg clutches in parthenogenetic species. It was shown that some parthenogenetic *Darevskia* lizards do not hatch from eggs. Studies of such embryos have shown multiple developmental anomalies [36–38].

### Z and W sex chromosomes in oocytes without genome duplication

Analysis of the behavior of the sex Z and W chromosomes in the hybrid karyotype of *D. armeniaca* revealed two variants of their localization during the pachytene and diplotene stages—joint and separate localization on the spread preparation of SCs (Figure 1A and B). Analysis of the nuclei with ZW joint localization revealed that the centromeric regions of the sex chromosomes were the most contiguous regions (Supplementary Figure SM1A). Thus, a pseudoautosomal region (PAR) is likely located in a pericentromeric region (Supplementary Materials Figure SM1). Nevertheless, the question of whether the true synapsis (homology in PAR) or Z–W association occurs remains open for unisexual forms of *Darevskia* [29].

Regardless of whether the Z and W sex chromosomes are located separately or nearby, Z and W univalents often form curved or even circular axial structures (Figure 1A and B). In general, the curved structure of sex univalents is characteristic of elongated asynaptic regions of sex chromosomes in different animals, especially in late prophase I, in diplotene [31, 39, 40]. Thus, the lengths of sex chromosomes in oocyte nuclei without endoduplication do not reflect the real lengths of univalents and do not allow us to accurately determine the position (according to length) of sex chromosomes in the karyotype (Figure 4A). On the other hand, we were able to measure the actual length of the WW sex bivalent (see the section below “WW bivalent formed *de novo* in the nuclei of oocytes after genomic endoduplication”).

### Meiotic prophase I in oocytes with genome endoduplication

The nuclei after genomic endoduplication found in the present study contained 38 bivalents (twice more than usual) at the pachytene and early diplotene stages (Figure 1C and D, Figure 2A–D). There are 36 autosomal pseudobivalents and two sex chromosome bivalents: ZZ and WW. All pseudobivalents are composed of identical copies of chromosomes due to premeiotic genome endoduplication.

The mechanism of premeiotic endoduplication, described for some unisexual animals of hybrid origin, is considered a way to avoid hybrid sterility [14]. Thus, unisexual reptile species can restore and maintain their 2n or 3n ploidy in many generations. Importantly, due to the identity of the chromosomes that are synapsing in the endoduplicated nuclei, crossing over does not lead to the emergence of new genetic combinations. Thus, our detection of normally loaded MLH1 protein in the pseudobivalents of endoduplicated nuclei (Figure 3B) is consistent with previous reports [8]. We can talk about the stopping of recombination [5, 41], a kind of “freezing” of the initial heterozygous state of the interspecific F1 hybrid.

An important marker in *Darevskia* karyotypes is a clearly visible heterochromatin block on the W chromosome (Figure 1C, D, and D′), which was previously described in mitotic metaphase plates [42, 43]. Since such a heterochromatin block is present only on one of the 38 bivalents (Figure 1C, D, and D′), this finding is another one confirmation of the assembly of pseudobivalents but not homeologous bivalents.

Interestingly, the m1 and m2 microchromosomes in the *D. armeniaca* hybrid karyotype inherited from the two parental species had different centromere positions clearly visible on the immunostained SC preparation (Figure 1D) and on the ideogram (Figure 4B). In oocytes after endoduplication, the corresponding SCs (m1m1 and m2m2) are formed without any shifting of the centromeres in bivalents, in addition confirming the assembly of the pseudobivalents (Figure 1D′). On the other hand, in the nonduplicated oocyte nuclei, we detected different variants of manifestation of the hom*e*ologous microbivalent: m1m2 SC without signs of disturbance (Supplementary Figure SM1A), possibly formed after synaptic adjustment [44–46] as well as located nearby (Figure 1A) or completely asynaptic m1 and m2 univalents (Supplementary Materials Figure SM1B).

### W‌W bivalent formed *de novo* in the nuclei of oocytes after genomic endoduplication

The temporary appearance of a second copy of the W chromosome in a germ cell line is, of course, an extraordinary fact. However, the ultrastructure of the synaptonemal complex allows normal assembly and synapsis of the newly emerged WW bivalent (Figure 1D′).

Notably, during the evolutionary period of existence of the gonochoristic maternal species (*D. mixta*), the W chromosome obviously did not have the possibility to synapse with its homolog to form WW synaptonemal complex, only with Z in a local pseudoautosomal region (PAR). This is a normal situation for the W and Y chromosomes in gonochoristic species. Rare cases of polysomy on the X and Y chromosomes are known in humans: 47, XXY; 47, XYY; 48, XXXY; 48, XYYY; 48, XXYY; 49, XXXXY; and 49, XXXYY [47]. On the other hand, rare cases of Y polysomy lead to severe developmental abnormalities and do not represent reproductive strategies.

We should separately note an interesting feature of the morphology of sex chromosomes (especially the W chromosome) in the nuclei of oocytes after premeiotic genome endoduplication. Usually, in animals with heteromorphic pairs of sex chromosomes, in meiotic prophase I, the sex chromosomes Z and W (or X and Y) have lengths that do not correspond to their chromosome numbers in the somatic karyotype. That is, the Z and W axial elements in meiotic prophase I are usually atypically elongated, curved, deformed, or coiled (Figure 1A and B). For this reason, a heteromorphic pair of sex chromosomes is often removed separately from autosomal pairs on karyotype diagrams [40, 48]. In contrast, in homogametic sex, such as in male reptiles, the sex Z chromosomes form a ZZ bivalent that is difficult or impossible to distinguish from autosomes [32, 35].

In the case of premeiotic duplication of chromosomes, the sex W chromosome is copied and can form a normal bivalent. We did not observe any deformations of axial elements in the structure of the WW bivalent in *D. armeniaca* (Figure 1D–D′). That is, in this case, the WW bivalent has a relevant length (not variable length usual for W, Z, X or Y univalents) since a normal synaptonemal complex is formed, similar to the usual ZZ bivalent in male reptiles. According to the constructed ideogram of the SC karyotype of endoduplicated nuclei, the WW bivalent had number 36 in the karyotype of *D. armeniaca* (Figure 4B).

Many studies have focused on reptilian ZW sex chromosomes in connection with the switch to unisexual reproduction [42, 43]. The W chromosome in lacertids is recognized as one of the most evolutionarily dynamic parts of the genomes [49]. Most often, these changes are characterized by a reduction in size, accumulation of heterochromatin, and tandem repeats (telomeric or satellite DNA) [43, 49, 50]. In Lacertidae, gradual transformation into the heterochromatic w sex microchromosome (Zw type) was suggested as an evolutionary trend [42, 43]. Recent bioinformatic analysis of Darevskia species revealed enrichment of the CLsat family of tandem DNA repeats in the pericentromeric regions of autosomes, especially in the W sex chromosome [51, 52].

### SC-tetravalents consisting of duplicated and homeologous chromosomes in the SC-karyotypes of *D. Armeniaca*

An interesting finding we made in the endoduplicated nuclei of *D. armeniaca* was the SC-tetravalents. We found fairly extensive ectopic synapsis at both the distal (Figure 2A) and centromeric (Figure 2B) ends of chromosomes, leading to the formation of SC-tetravalents in nuclei after genome endoduplication. Similar unique cases have been described in mammalian meiosis [53–56]. It is likely that in *Darevskia* hybrid unisexual species such configurations arise between corresponding homeologs. Apparently, during the assembly of SCs in the endoduplicated nucleus of *D. armeniaca* oocytes, active processes associated with the correction of ectopic synapsis occur. Such synaptic resolving processes were previously described for many polyploids [57–61], and for the competitive synapsis between unpaired regions (univalents) of XY sex bivalents and autosomes in male mammals [62]. These studies showed that non-homologous partial synapsis often occurs in zygotene in the pericentromeric/peritelomeric regions of chromosomes and can be corrected in subsequent stages of meiotic prophase I. Indeed, it is known that such synaptic associations can be resolved before metaphase I and do not affect normal chromosome segregation [57, 62].

### Premeiotic genome endoduplication and postmeiotic ploidy restoration pathways

An important question is, after the discovery of premeiotic genome endoduplication in *D. armeniaca*, as the mechanism of ploidy restoration, can we reject the possibility of a postmeiotic automictic mechanisms through central fusion (the fusion of the oocyte and the first polar body) and post-meiotic genome duplication [29, 63]? The mechanism of premeiotic endoduplication ensures long-term preservation of heterozygosity of the unisexual form. On the other hand, in numerous nuclei without chromosome duplication, we detected homo*e*ologous synapsis for both unisexual species examined: *D. unisexualis* [24, 29] and *D. armeniaca* (Figure 1A and B; Supplementary Material Figures SM1A, B). Moreover, we showed loading of the MLH1 protein associated with crossing over into bivalents of homeologues (Figure 3A) and [24]. Finally, we previously revealed weak meiotic checkpoints in *Darevskia* hybrid individuals, allowing even triploid males to produce numerous mature but aneuploid spermatids [19].

Thus, there is a probability that oocytes without endoduplication can overcome the synaptic checkpoints of meiosis in parthenogenetic *Darevskia*. However, what happens next to these oocytes is unknown. If natural populations of unisexual *Darevskia* species contain individuals born as a result of a central fusion or post-meiotic genome duplication mechanisms, then we should expect deviations from strict clonality and some polymorphism in the populations. According to some studies, all unisexual species of *Darevskia* demonstrate some diversity in their morphology and karyotypes. This issue requires detailed analysis in the future using genomic approaches.

### Developmental deviation in parthenogenetic species of the genus *Darevskia*

Numerous findings of embryonic abnormalities have been documented for parthenogenetic *Darevskia* rock lizards [36–38]. Data from previous studies indicate that these developmental deviations (up to 10 different types) occur quite often (3–6.8%) in unisexual species of the genus *Darevskia* (4.5–4.9% for *D. armeniaca*) compared to gonochoristic species (1.1–1.8%) [38].

Two recorded by us cases of embryos alive but unable to hatch from eggs (Supplementary Material Figure SM3А, 3B) are consistent with previous findings of developmental embryonic abnormalities described in the classification of I.S. Darevsky: unclosed body cavities, complete absence of the lower jaw, disproportion and curvature of the jaws [36, 38]. Relatively similar findings of developmental deviations were recently described in the genus *Aspidoscelis* and are associated with facultative parthenogenesis [63]. These data may indicate a high risk of errors in oogenesis during the process of endoduplication. Further comparative studies of the karyotypes of such individuals may help to understand whether errors occurred in meiosis. Overall, an important direction for future work is to investigate the increased risks of non-viable embryo formation during ploidy restoration in parthenogenetic *Darevskia*.

## Supplementary Material

Supplementary_Materials_ver_11_ioaf091

## Data Availability

The data discussed in this publication have been deposited online: Spangenberg, V. (2024). Zenodo. doi:10.5281/zenodo.10891208
