# Supplementary material for: Tendency towards clonality: deviations of meiosis in parthenogenetic Caucasian rock lizards
Source: Biol Reprod. 2025 May 22;113(2):387–96. doi: 10.1093/biolre/ioaf091 (PMC12358248; doi:10.1093/biolre/ioaf091)
Supplement: Supplementary_Materials_ver_11_ioaf091 [file supplementary_materials_ver_11_ioaf091.docx]

**Supplementary Materials**


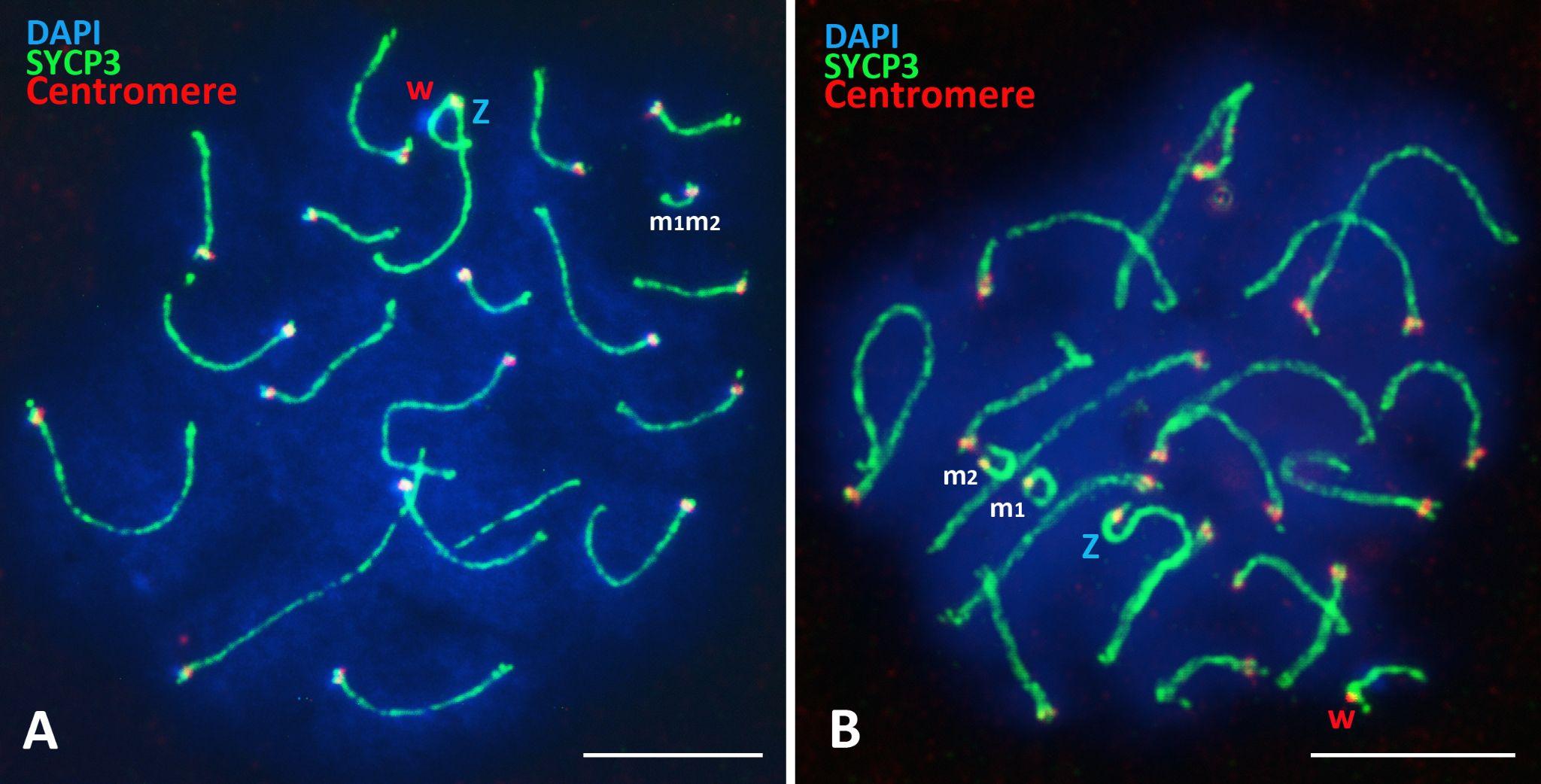


**Figure SM 1**. early diplotene nucleus of normal ploidy with 18 autosomal homeologous bivalents and sex ZW-bivalent. The Z and W univalents contact in their centromeric regions. Heterochromatic region on the W chromosome is clearly visible. Axial elements are immunostained with anti-SYCP3 antibodies (green), сentromeres with ACA-antibodies (red), chromatin stained with DAPI, 4′,6-diamidino-2-phenylindole (blue). Bars: 10 μm.


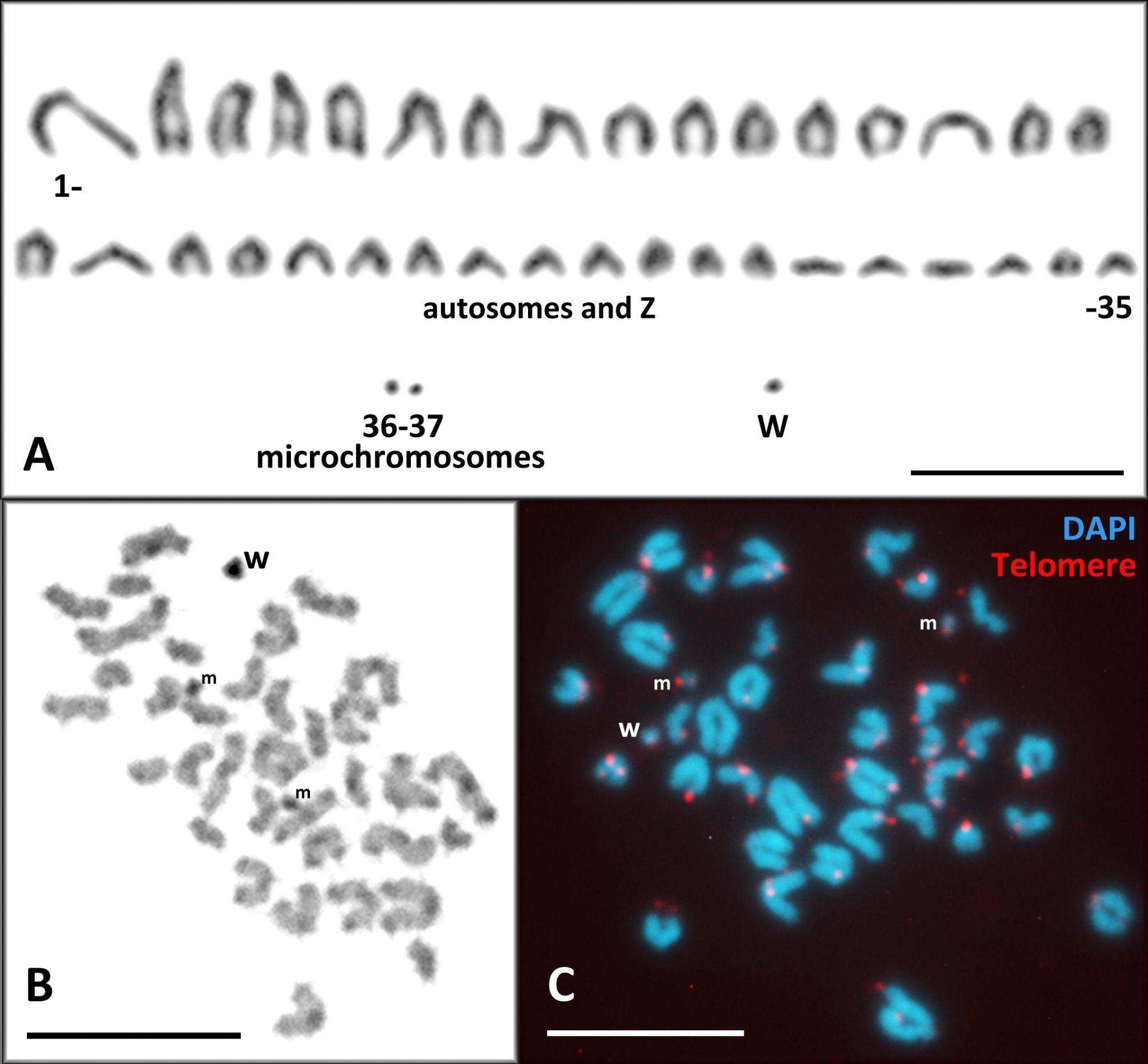


**Figure SM 2.** Mitotic karyotypes of *D. armeniaca* after conventional Giemsa staining (A), C-banding (B), and FISH with DNA probe to telomere repeats (C). Karyotype has 34 autosomal acrocentric chromosomes, 2 microchromosomes, Z chromosome (that is indistinguishable from autosomes) and W chromosome that has strong heterochromatin block detectable by C-banding and no extra accumulation of telomere repeats. Bars: 10 μm.


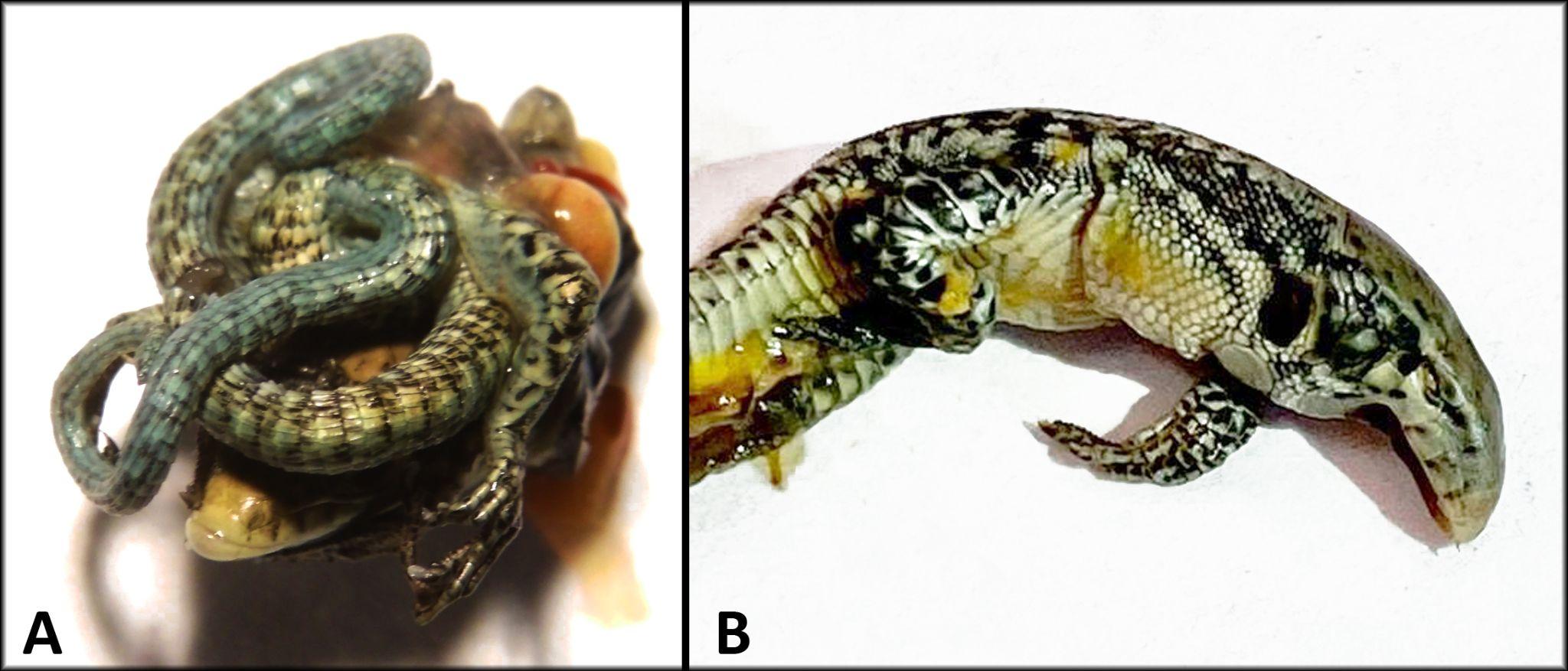


**Figure SM 3**. Detected by us embryonic developmental anomalies during incubation of eggs of parthenogenetic species *D. armeniaca*. A - unclosed body cavities; B - complete absence of the lower jaw, the deviations known from previous publications.
